# Performance of Current Diagnostic Tools in Detecting Latent Tuberculosis Among Healthcare Workers: A Systematic Review

**DOI:** 10.7759/cureus.70621

**Published:** 2024-10-01

**Authors:** Aishwarya R, Maheshwary D, Leela KV, Vijay R Suriya, Kanya R

**Affiliations:** 1 Microbiology, SRM Medical College Hospital and Research Centre, SRM Institute of Science and Technology, Chengalpattu, IND

**Keywords:** clia-igra, diagnostic test accuracy, healthcare worker, igra, latent tb, latent tuberculosis infection, quantiferon gold plus, quantiferon-tb, tb screening, tst

## Abstract

Testing for latent tuberculosis infection is essential for diagnosing *Mycobacterium tuberculosis* infections in asymptomatic individuals. Preventing the transition of latent to active tuberculosis is imperative, especially in high-risk populations such as healthcare workers. Interferon-gamma release assays (IGRAs) and the Mantoux/tuberculin skin test (TST) are two examples of diagnostic instruments utilized for detection. Systematic evaluations of the characteristics of widely available tests are very helpful for diagnosticians because these tests might not be easily accessible in situations with limited resources. This systematic review aims to evaluate and compare the diagnostic accuracy of tests for latent tuberculosis infection in healthcare workers. The review, conducted from 2013 to 2024, aimed to identify studies on “Latent Tuberculosis,” “Healthcare workers,” “Diagnostic modalities,” “TST,” “Interferon-gamma release assays,” and “IGRA.” The review followed Preferred Reporting Items for Systematic Reviews and Meta-Analyses 2020 guidelines and developed a data extraction toolkit. Three authors independently reviewed the literature, ensuring uniformity. Discrepancies were resolved through discussions and mediation until a consensus was reached. Statistical significance was defined as a p-value of 0.05 or lower. The review provides valuable insights into the diagnostic accuracy of these tests, particularly in high-risk populations. The TST had a sensitivity of 76.5% (confidence interval (CI) = 61.5-91.5%) and specificity of 77.2% (CI = 65-85%). Its positive predictive value (PPV) was 54.8% (CI = 45-65%), and the negative predictive value (NPV) was 88.5% (CI = 85-92%), with an odds ratio of 63.6 and an area under the curve (AUC) of 0.72. QuantiFERON-TB Gold In-Tube (QFT-GIT) showed a sensitivity of 68.35% (CI = 67.15-70.55%) and a specificity of 82.32% (CI = 72.32-97.47%). Its PPV was 56% (CI = 54-92%), and NPV was 92.7% (CI = 89-96%), with an odds ratio of 357.9 and an AUC of 0.767. QuantiFERON-TB Gold Plus (QFT-Plus) had a sensitivity of 85% (CI = 78.9-91.1%) and a specificity of 73.52% (CI = 35.71-93.75%). Its PPV was 59.2% (CI = 39.3-78.2%), and NPV was 95% (CI = 91.2-98%), with an odds ratio of 125.39 and an AUC of 0.89. T-SPOT.TB showed a sensitivity of 92% (CI = 87-97%) and a specificity of 95.7% (CI = 94-98%). Its PPV was 86.8% (CI = 85-95.8%), and NPV was 95.8% (CI = 92.5-96.7%), with an odds ratio of 1.03 and an AUC of 0.7. CLIA-IGRA had a sensitivity of 100% (CI = 99.9-100%) and a specificity of 95.57% (CI = 95.57-100%). Its PPV was 96.8% (CI = 96.8-100%), and NPV was 99.8% (CI = 99.8-100%), with an odds ratio of 1509 and an AUC of 0.97. HBHA-induced IGRA showed a sensitivity of 86.4% (CI = 71.1-97.3%) and a specificity of 82.5% (CI = 66.4-92.6%). Its PPV was 86.8% (CI = 66.7-95.3%), and NPV was 86.4% (CI = 57.8-95.7%), with an odds ratio of 6.18 and an AUC of 0.886. There are specific benefits and drawbacks of each diagnostic test for latent tuberculosis infection. With its exceptional sensitivity and specificity, the CLIA-IGRA test is a top choice for a precise diagnosis of tuberculosis. Practical factors such as availability and cost, however, might prevent its widespread usage. Both the QuantiFERON and TST are still useful tools, especially when used in certain populations or situations when their performance characteristics meet clinical requirements.

## Introduction and background

As they are more likely to come into contact with *Mycobacterium tuberculosis*, healthcare workers are at a major occupational risk from latent tuberculosis infection, particularly in low- and middle-income countries where tuberculosis prevalence is high, where over than 1.7 billion people or around 22% of the global population are estimated to be infected [1]. Accurate and timely identification of latent tuberculosis among healthcare workers is crucial for preventing the progression to active tuberculosis and for implementing appropriate public health measures. The traditional Mantoux/tuberculin skin test (TST) has been widely used for latent tuberculosis diagnosis, but it has limitations, including environmental mycobacteria and the Bacille Calmette-Guérin (BCG) immunization cross-reactivity [2]. The development of interferon-gamma release assays (IGRAs) and their growing application in clinical practice are responses to these constraints [3].

Modern developments in molecular diagnostics have resulted in the creation of novel in-vitro tests quantifying the amount of interferon-gamma (INF-γ) that T cells secrete that have been sensitized to *M. tuberculosis* antigens [4]. Due to the inclusion of antigens not found in BCG vaccination strains or common non-tuberculous mycobacteria (NTM) species like *M. avium*, the aforementioned tests show increased specificity than the TST. In low-incidence areas, IGRAs provide a more robust prediction value for the development of latent tuberculosis to active tuberculosis in close contacts, in addition to having higher specificity and sensitivity than the TST. Additionally, IGRAs correlate better with surrogate indicators of exposure to *M. tuberculosis* in low tuberculosis prevalence areas [5].

The risk of latent tuberculosis infection among healthcare workers is considerably elevated due to frequent exposure to infectious patients. As a result, effective screening and diagnostic strategies are crucial to mitigate this risk and prevent the progression to active tuberculosis [5,6]. As there is no reference standard to diagnose latent tuberculosis, a study like this evaluating various outcomes from different studies has to be evaluated to provide insights into how to proceed with appropriate investigations when it comes to latent tuberculosis [7]. IGRAs, such as the QuantiFeron-TB Gold In-Tube (QFT-GIT) and the newer QuantiFeron-TB Gold Plus (QFT-Plus), measure the release of INF-γ in response to *M. tuberculosis*-specific antigens and are not affected by BCG vaccination [8].

The objective of this systematic study is to assess and contrast the effectiveness and value of various latent tuberculosis diagnostic modalities among healthcare workers. The review will encompass studies on TST, QFT-GIT, QFT-Plus, and T-SPOT.TB, focusing on their sensitivity, specificity, and operational feasibility in high tuberculosis burden settings. By looking closely at all of these diagnostic tools, we hope to come up with evidence-based suggestions for latent tuberculosis screening in healthcare settings [9]. This will help healthcare workers better control and avoid tuberculosis [10]. This review aims to systematically evaluate the efficacy and reliability of these diagnostic methods among healthcare workers.

## Review

Methodology

An elaborate search was performed on databases such as PubMed, Cochrane Library, EMBASE, Web of Science, Google Scholar, and Scopus from 2013 to July 2024. Keywords including “Latent Tuberculosis” AND “Healthcare workers” AND “Diagnostic modalities” AND “TST” OR “IGRA” OR “Interferon Gamma Release assay” OR “QuantiFERON-TB” OR “QuantiFERON-TB Gold In-Tube” were used. Articles were chosen from all countries, and all diagnostic tests were considered. A systematic review of the literature was conducted in compliance with the Preferred Reporting Items for Systematic Reviews and Meta-Analyses (PRISMA) 2020 guidelines.

Selection Criteria

Inclusion criteria: The review included studies that focused on the diagnostic accuracy, sensitivity, and specificity of TST and IGRAs among healthcare workers. Studies included in the review were prevalence and observational (cohort, case-control, and cross-sectional) and were conducted in countries with both low and high tuberculosis incidence. The parameters of QFT-GIT, QFT-Plus, and TST were analyzed categorically.

Exclusion: Studies that did not target healthcare professionals and did not report on the performance characteristics of the above-mentioned tests were excluded.

Extraction Toolkit: A toolkit was developed to extract data on key elements such as the first author, publication year, study design, sample size, and detection methods, including their sensitivity and specificity. To ensure uniformity in screening criteria and data collection, three authors independently reviewed the literature and collected the necessary information. Any discrepancies were resolved through detailed discussions and the involvement of a mediator until a consensus was reached.

Statistical Analysis and Data Integration

Using SPSS version 29 (IBM Corp., Armonk, NY, USA), we assessed various metrics such as positive predictive value (PPV), negative predictive value (NPV), sensitivity, specificity, accuracy, and diagnostic odds ratio. Furthermore, we generated 95% confidence intervals (CIs) and plotted them against their area under the curve (AUC) for the summary receiver operating characteristic (SROC) curves for the TST, QFT-Plus, QFT-GIT, and T-Spot TB tests. Statistical significance was defined as a p-value of 0.05 or lower. Quality analysis of the included articles [1-36] was done to understand and summarize the various parameters considered before inclusion (Table 1).

**Table 1:** Quality assessment of included studies.

Authors	Theoretical approach	Study design	Did the study have a rigorous search methodology?	Were the methods reliable?	Was there a reference standard for comparison?	Is the analysis reliable?	Are the findings convincing?	Are the conclusions adequate?	Overall quality assessment
Tang et al. [27]	Clear	Cross-sectional	Yes	Yes	Yes	Yes	Yes	Yes	Good quality
Hung et al. [8]	Clear	Case-control	Yes	Yes	Yes	Yes	Yes	No	Average quality
Whitaker et al. [29]	Clear	Retrospective cohort	Yes	Yes	Yes	Yes	Yes	Yes	Good quality
Keshavarz et al. [11]	Clear	Longitudinal	No	Yes	Yes	Yes	Yes	No	Good quality
Rudeeaneksin et al. [16]	Clear	Cross-sectional	Yes	Average	Yes	Yes	Yes	Yes	Good quality
Agaya et al. [20]	Clear	Case-control	Yes	Yes	No	Yes	Yes	Yes	Good quality
Sabri et al. [30]	Clear	Case-control	Yes	Yes	Yes	Average	Yes	Average	Average quality
Mostafavi et al. [31]	Clear	Cross-sectional	Yes	Yes	Yes	Yes	Yes	Yes	Good quality
Aksornchindarat et al [21]	Clear	Retrospective cohort	Yes	Yes	Yes	Yes	Yes	Yes	Good quality
Kim et al. [24]	Clear	Cross-sectional	Yes	Yes	Yes	Average	Yes	Yes	Average quality
Hsieh et el. [32]	Clear	Cross-sectional	Yes	Yes	Yes	Yes	Yes	Yes	Good quality
Islam et al. [12]	Clear	Cross-sectional	Yes	Yes	Yes	Yes	Yes	Yes	Good quality
Park et al. [18]	Clear	Cross-sectional	Yes	Average	No	Yes	Yes	Yes	Average quality
Lina Yi et al. [19]	Clear	Retrospective cohort	Yes	Yes	Yes	Yes	Yes	Yes	Good quality
Gocmen et al. [33]	Clear	Case-control	No	Yes	Yes	Yes	Yes	No	Average quality
Apriani et al. [34]	Clear	Longitudinal	Yes	Yes	Yes	Yes	Yes	Yes	Good quality
Al Hajoj et al. [35]	Clear	Cross-sectional	Yes	Yes	Yes	Yes	Yes	Yes	Good quality
Schablon et al. [13]	Clear	Retrospective cohort	Yes	Yes	Yes	Average	Yes	Average	Average quality
Erawati et al. [17]	Clear	Case-control	Yes	Yes	Yes	Yes	Yes	No	Good quality
Graves et al. [36]	Clear	Cross-sectional	Yes	Yes	Yes	Yes	Yes	Yes	Good quality

Results

Summary of Eligible and Incorporated Research

**Figure 1:**
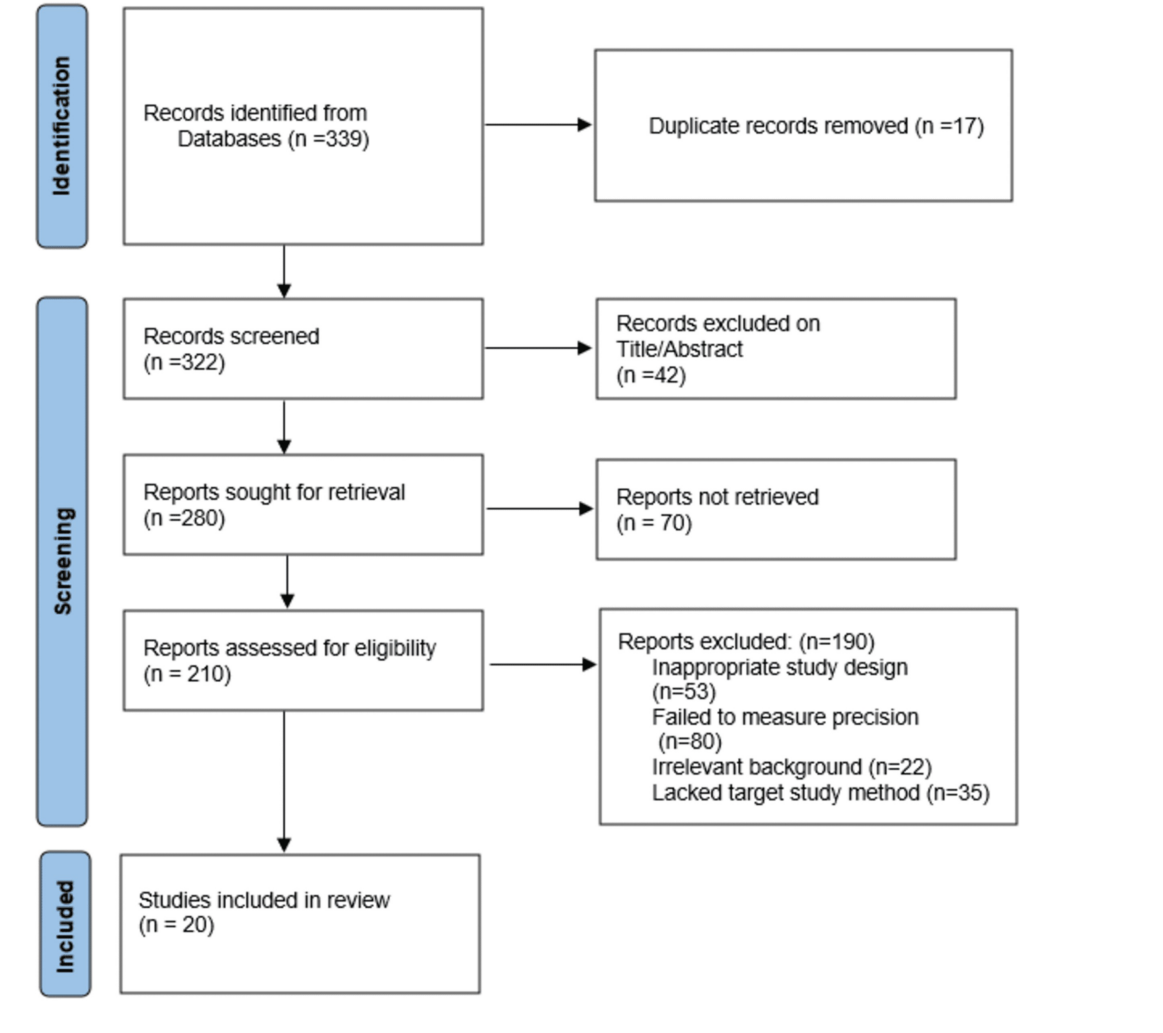
Preferred Reporting Items for Systematic Reviews and Meta-Analyses (PRISMA) flowchart for retrieval of included articles.

Through meticulous searching and analysis, 339 articles were identified from 2013 to July 2024 and screened. Based on the abstract and author credits, 17 duplicates were removed. Overall, 322 underwent initial screening, of which 42 were excluded based on abstract and title match. Subsequently, 280 articles were assessed for eligibility, of which 190 articles were excluded based on the wrong study design, not measuring the precision of the study, lacking the target method, and irrelevant background. Finally, 20 articles were included for analysis that focused on QFT-Plus, QFT-GIT, TST, and T-Spot TB. Quality assessment was done based on the type of study, parameters assessed, and tools used. An assessment was done to grade it as good or average quality based on the criteria provided by the PRISMA 2020 checklist included in the Appendix. The PRISMA flowchart is shown in Figure 1.

Assessment of Diagnostic Efficacy of the Tests

The TST had a sensitivity of 76.5% (CI = 61.5-91.5%) and a specificity of 77.2% (CI = 65-85%). Its PPV was 54.8% (CI = 45-65%), and NPV was 88.5% (CI = 85-92%), with an odds ratio of 63.6 and an AUC of 0.72. QFT-GIT showed a sensitivity of 68.35% (CI = 67.15-70.55%) and a specificity of 82.32% (CI = 72.32-97.47%). Its PPV was 56% (CI = 54-92%), and NPV was 92.7% (CI = 89-96%), with an odds ratio of 357.9 and an AUC of 0.767. QFT-Plus had a sensitivity of 85% (CI = 78.9-91.1%) and a specificity of 73.52% (CI = 35.71-93.75%). Its PPV was 59.2% (CI = 39.3-78.2%), and NPV was 95% (CI = 91.2-98%), with an odds ratio of 125.39 and an AUC of 0.89. T-SPOT.TB showed a sensitivity of 92% (CI = 87-97%) and a specificity of 95.7% (CI = 94-98%). Its PPV was 86.8% (CI = 85-95.8%), and NPV was 95.8% (CI = 92.5-96.7%), with an odds ratio of 1.03 and an AUC of 0.7. CLIA-IGRA had a sensitivity of 100% (CI = 99.9-100%) and a specificity of 95.57% (CI = 95.57-100%). Its PPV was 96.8% (CI = 96.8-100%), and NPV was 99.8% (CI = 99.8-100%), with an odds ratio of 1,509 and an AUC of 0.97. HBHA-induced IGRA showed a sensitivity of 86.4% (CI = 71.1-97.3%) and a specificity of 82.5% (CI = 66.4-92.6%). Its PPV was 86.8% (CI = 66.7-95.3%), and NPV was 86.4% (CI = 57.8-95.7%), with an odds ratio of 6.18 and an AUC of 0.886 (Table 2).

**Table 2:** Comparative performance metrics of various diagnostic tests for tuberculosis. TB = tuberculosis; TST = tubercullin skin test; IGRA = interferon-gamma release assay; PPV = positive predictive value; NPV = negative predictive value; AUC = area under the curve; HBHA = heparin-binding hemagglutinin; CLIA = chemiluminescence immunoassay

Test	Sensitivity (95% CI)	Specificity (95% CI)	PPV (95% CI)	NPV (95% CI)	Odds ratio	AUC
Tuberculin Skin Test (TST)	76.5% (61.5-91.5%)	77.2% (65-85%)	54.8% (45-65%)	88.5% (85-92%)	63.6	0.72
QuantiFERON-GIT	68.35% (67.15-70.55%)	82.32% (72.32-97.47%)	56% (54-92%)	92.7% (89-96%)	357.9	0.767
QuantiFERON-TB Gold Plus	85% (78.9-91.1%)	73.52% (35.71-93.75%)	59.2% (39.3-78.2%)	95% (91.2-98%)	125.39	0.89
T-Spot TB	92% (87-97%)	95.7% (94-98%)	86.8% (85-95.8%)	95.8% (92.5-96.7%)	1.03	0.7
CLIA-IGRA	100.0% (99.9-100%)	95.57% (95.57-100%)	96.8% (96.8-100%)	99.8% (99.8-100%)	1509	0.97
HBHA-Induced IFN-γ Assay	86.4% (71.1-97.3%)	82.5% (66.4-92.6%)	86.8% (66.7-95.3%)	86.4% (57.8-95.7%)	6.18	0.886

**Figure 2:**
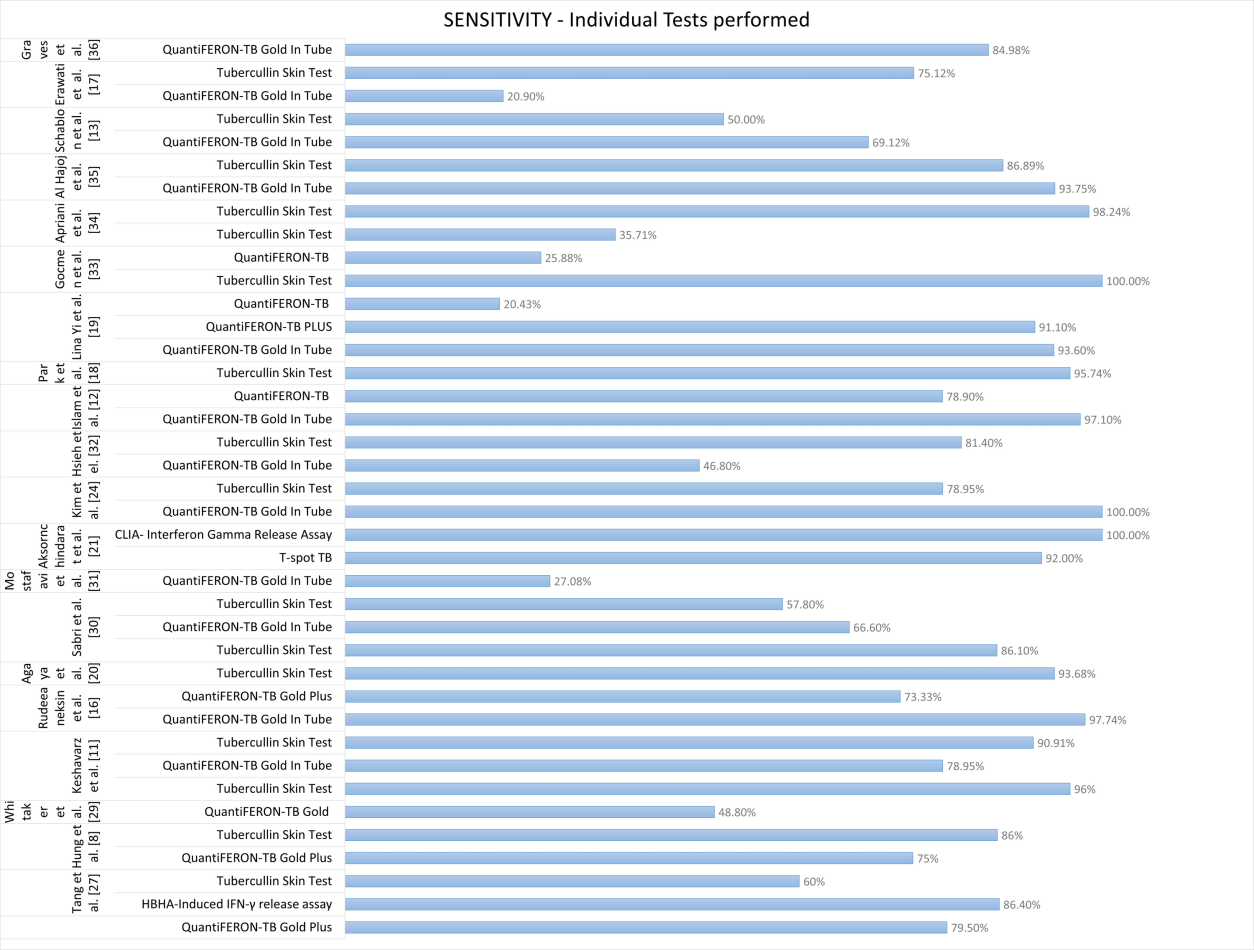
Performance characteristics: specificity of QuantiFeron TB Plus, QuantiFeron TB GIT, TST and T-Spot TB, CLIA, and HBHA-IGRA. TB = tuberculosis; GIT = gold in tube; TST = tubercullin skin test; IGRA = interferon-gamma release assay; PPV = positive predictive value; NPV = negative predictive value; HBHA = heparin-binding hemagglutinin; CLIA = chemiluminescence immunoassay

An overall comparison of sensitivity as a performance characteristic is illustrated in Figure 2.

**Figure 3:**
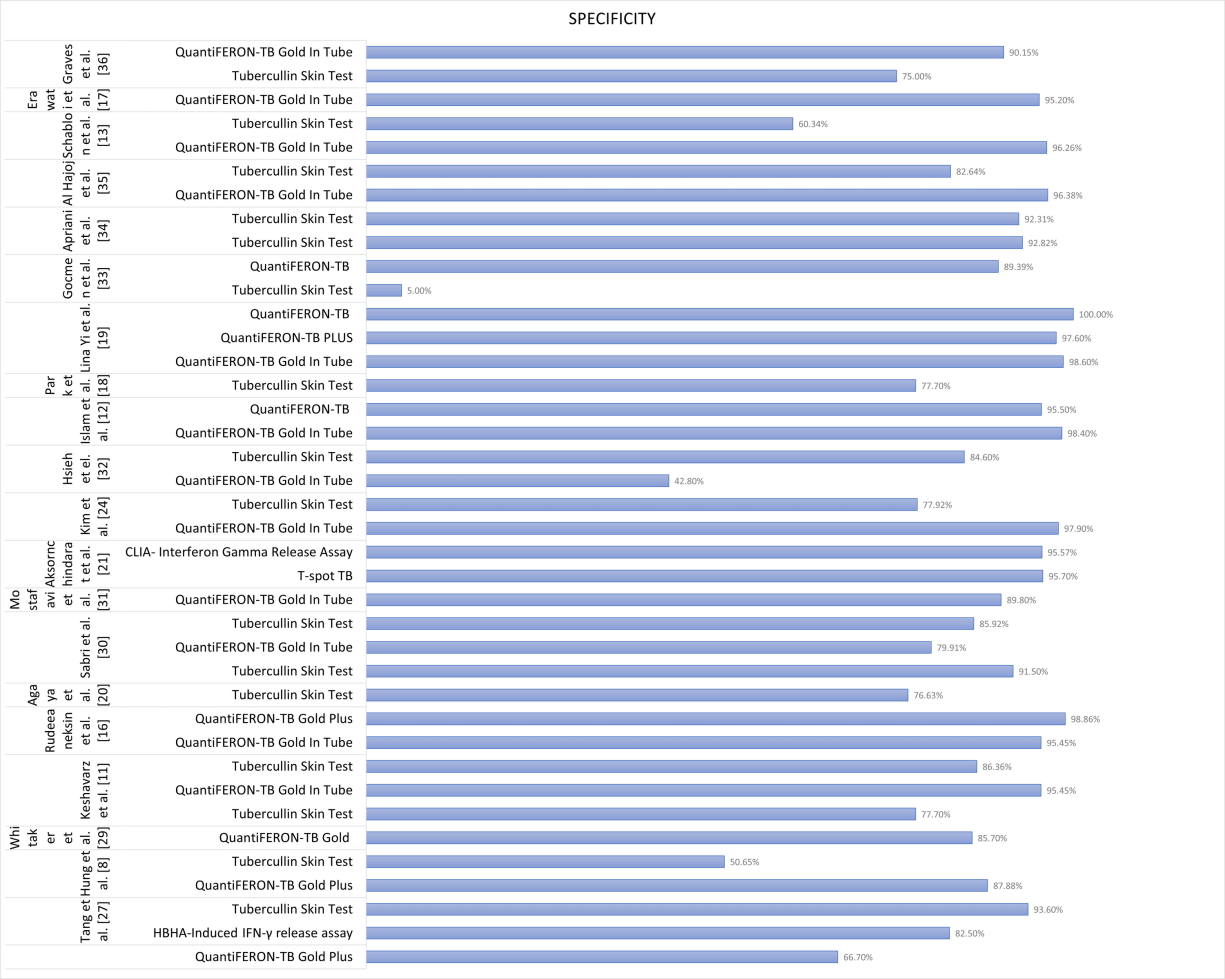
Performance characteristics: specificity of QuantiFeron TB Plus, QuantiFeron TB GIT, TST and T-Spot TB, CLIA, and HBHA-IGRA. TB = tuberculosis; GIT = gold in tube; TST = tubercullin skin test; BCG = Bacille Calmette-Guérin; IGRA = interferon-gamma release assay; HBHA = heparin-binding hemagglutinin; CLIA = chemiluminescence immunoassay

An overall comparison of specificity as a performance characteristic is illustrated in Figure 3.

**Figure 4:**
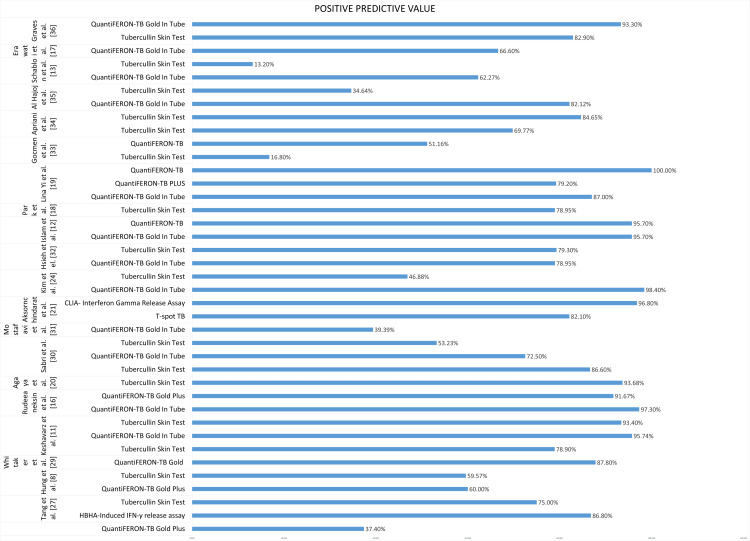
Performance characteristics: positive predictive value of QuantiFeron TB Plus, QuantiFeron TB GIT, TST and T-Spot TB, CLIA, and HBHA-IGRA. TB = tuberculosis; GIT = gold in tube; TST = tubercullin skin test; BCG = Bacille Calmette-Guérin; IGRA = interferon-gamma release assay; HBHA = heparin-binding hemagglutinin; CLIA = chemiluminescence immunoassay

An overall comparison of PPV as a performance characteristic is illustrated in Figure 4.

**Figure 5:**
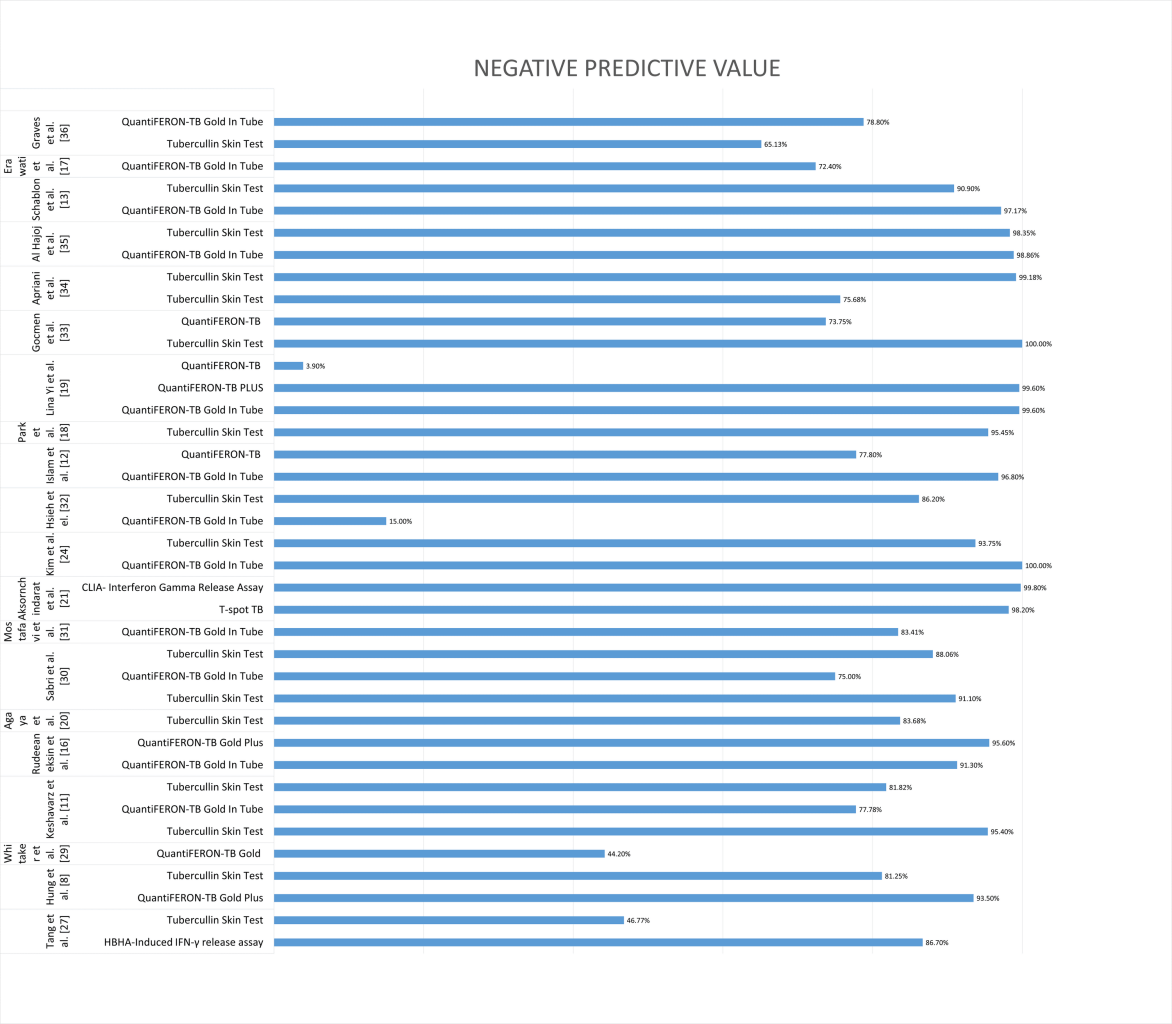
Performance characteristics: negative predictive value of QuantiFeron TB Plus, QuantiFeron TB GIT, TST and T-Spot TB, CLIA, and HBHA-IGRA. TB = tuberculosis; GIT = gold in tube; TST = tubercullin skin test; BCG = Bacille Calmette-Guérin; IGRA = interferon-gamma release assay; HBHA = heparin-binding hemagglutinin; CLIA = chemiluminescence immunoassay

An overall comparison of NPV as a performance characteristic is illustrated in Figure 5.

**Figure 6:**
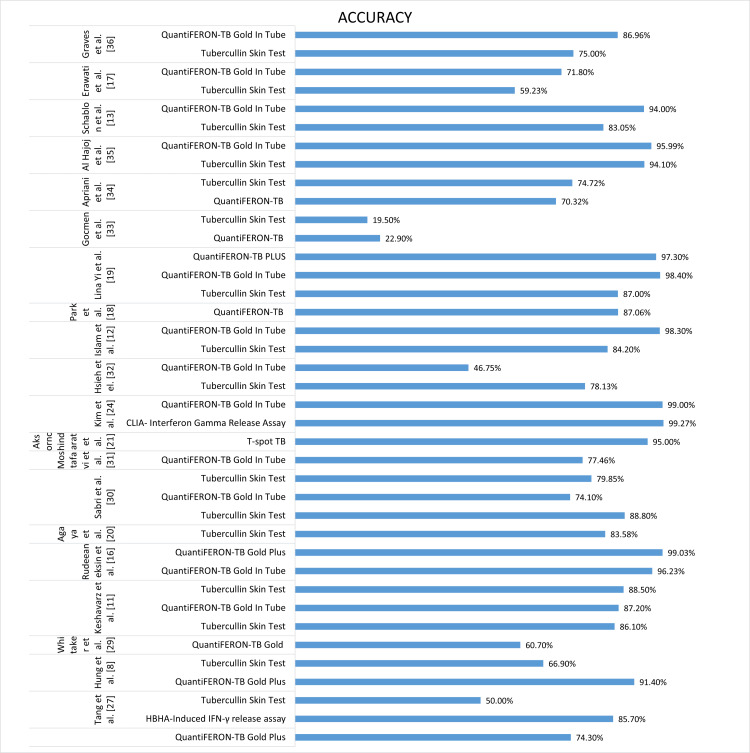
Performance characteristics: accuracy of QuantiFeron TB Plus, QuantiFeron TB GIT, TST and T-Spot TB, CLIA, and HBHA-IGRA. TB = tuberculosis; GIT = gold in tube; TST = tubercullin skin test; BCG = Bacille Calmette-Guérin; IGRA = interferon-gamma release assay; HBHA = heparin-binding hemagglutinin; CLIA = chemiluminescence immunoassay

An overall comparison of accuracy as a performance characteristic is illustrated in Figure 6.

**Figure 7:**
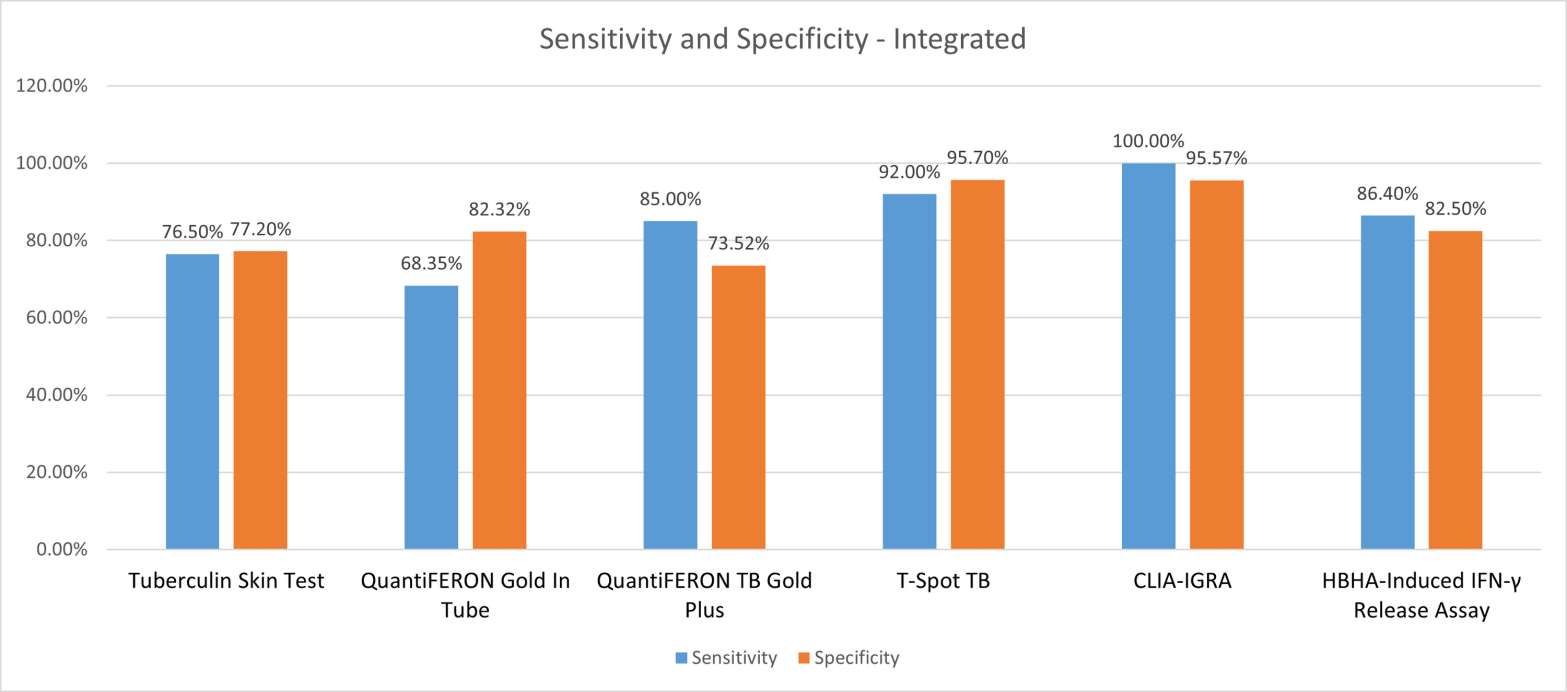
Performance characteristics – Integrated Sensitivity, Specificity of QuantiFeron TB Plus, QuantiFeron TB GIT, TST and T-Spot TB, CLIA and HBHA-IGRA TB: Tuberculosis; HBHA-Heparin-Binding Hemagglutinin; CLIA- Chemiluminescent Immunoassay

An integrated comparison of sensitivity and specificity among all the studies included is depicted in Figure 7.

**Figure 8:**
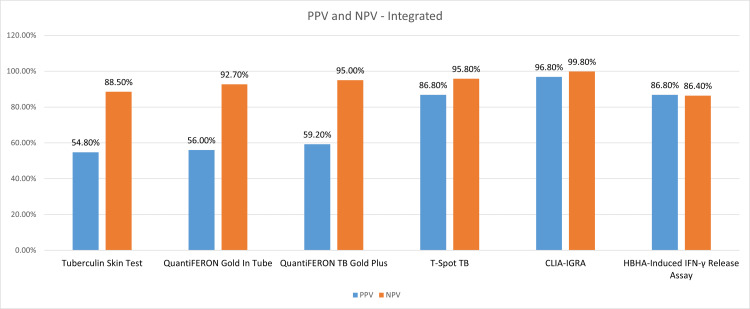
Performance characteristics: integrated PPV and NPV of QuantiFeron TB Plus, QuantiFeron TB GIT, TST and T-Spot TB, CLIA, and HBHA-IGRA. TB = tuberculosis; HBHA = heparin-binding hemagglutinin; CLIA = chemiluminescent immunoassay; PPV = positive predictive value; NPV = negative predictive value

An integrated comparison of PPV and NPV among all the studies included is depicted in Figure 8.

**Figure 9:**
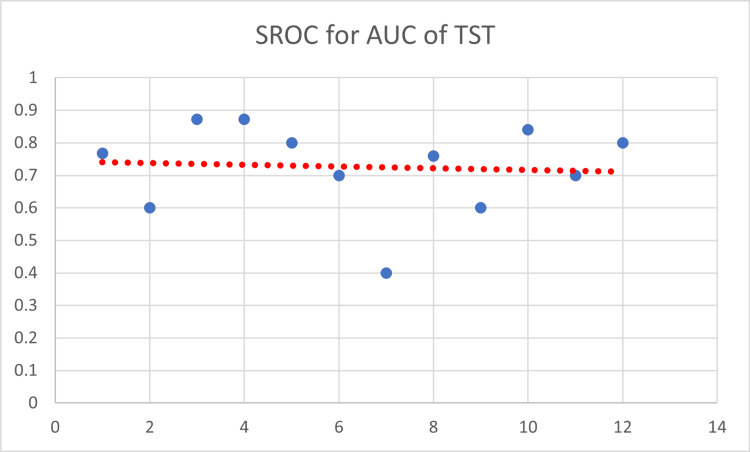
Summary receiver operating characteristic curve of TST. SROC = summary receiver operating characteristic curve; AUC = area under the curve; TST = tuberculin skin test

The SROC of TST as a single characteristic is depicted in Figure 9.

**Figure 10:**
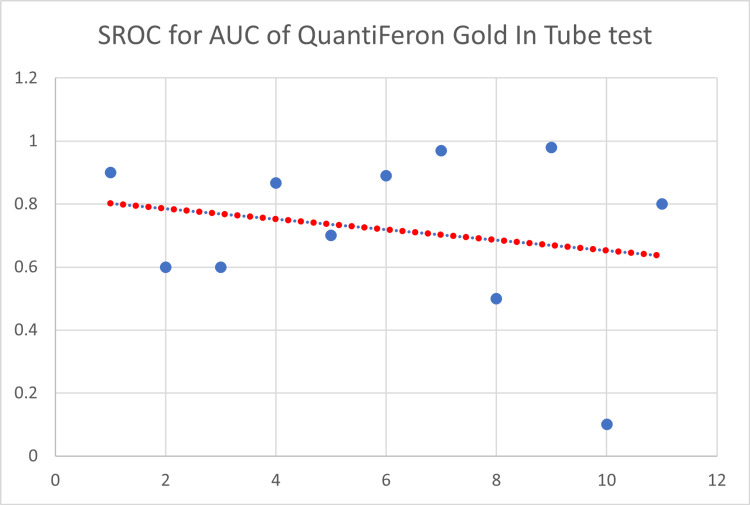
Summary receiver operating characteristic curve for QuantiFeron GIT. SROC = summary receiver operating characteristic curve; AUC = area under the curve

The SROC of QFT-GIT as a single characteristic is depicted in Figure 10.

**Figure 11:**
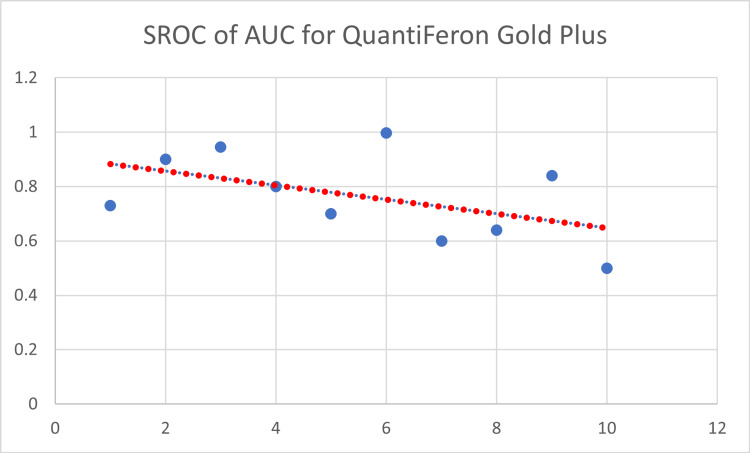
Summary receiver operating characteristic curve of QuantiFeron Gold Plus. SROC = summary receiver operating characteristic curve; AUC = area under the curve

The SROC of QFT-Plus as a single characteristic is depicted in Figure 11.

**Figure 12:**
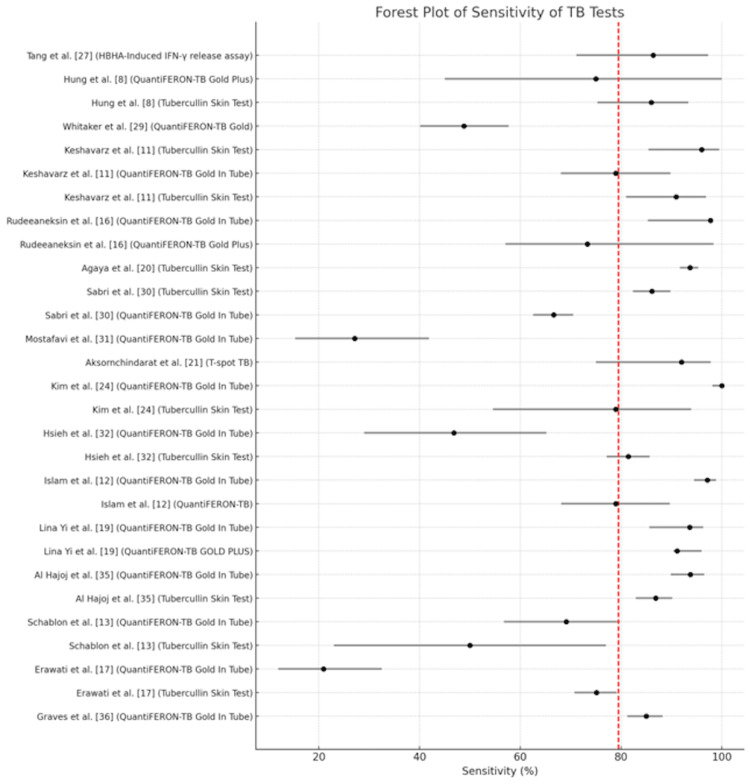
Forest plot illustrating the cumulative performance of the tests in terms of sensitivity with 95% confidence intervals.

**Figure 13:**
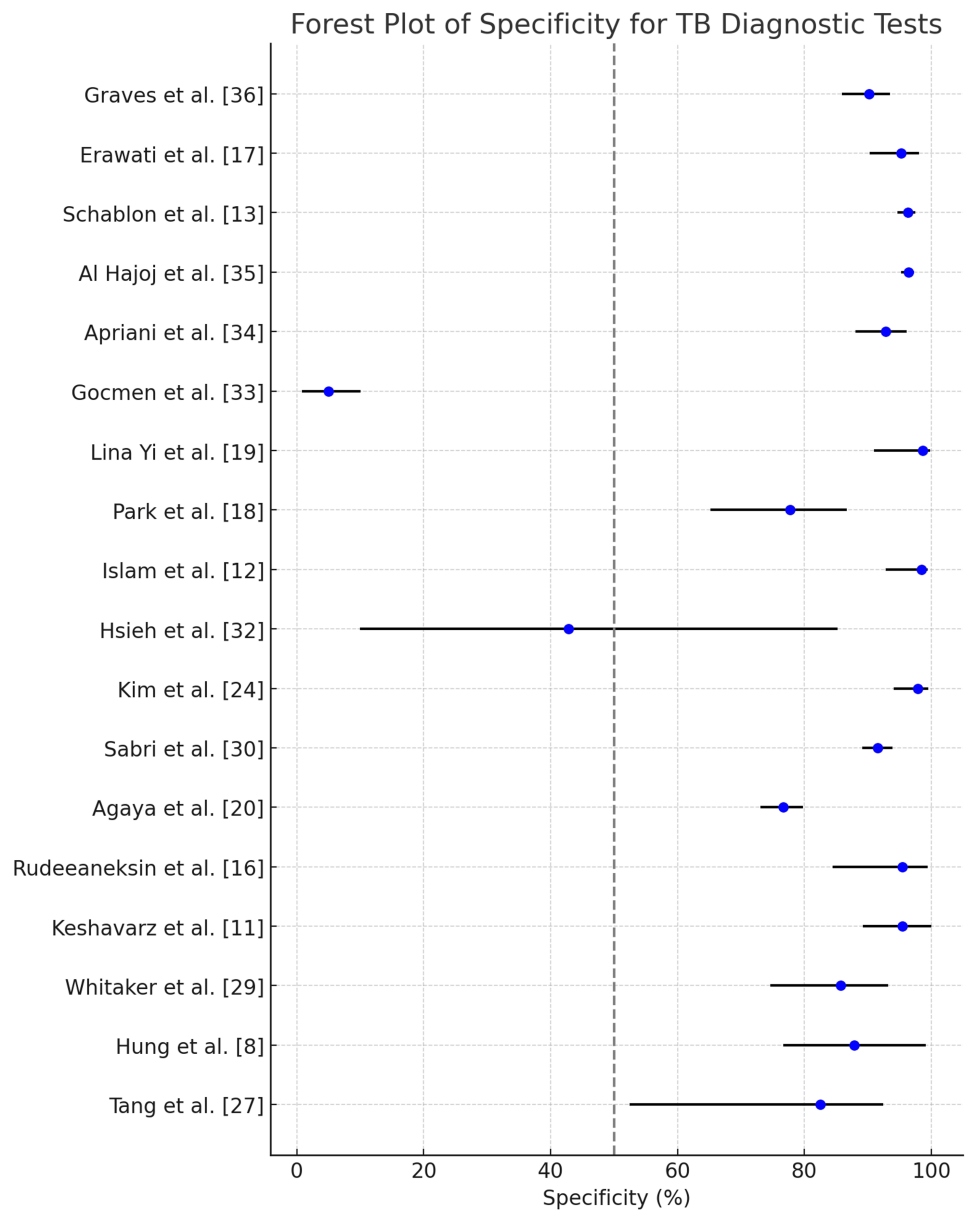
Forest plot illustrating the cumulative performance of the tests in terms of specificity with 95% confidence intervals.

A forest plot enumerating the sensitivity of all the studies analyzed including confidence intervals is depicted in Figure 12. A forest plot enumerating the specificity of all the studies analyzed including confidence intervals is depicted in Figure 13.

Discussion

To diagnose latent tuberculosis a range of diagnostic tests, each with varying principles, is needed. This discussion elaborates on the effectiveness of several prominent tests based on their performance characteristics.

Tuberculin Skin Test

The TST/Mantoux test remains a widely used diagnostic tool for latent tuberculosis [10,11]. Its sensitivity varies significantly across studies, ranging from 60% to 96%, with the broader range reflecting variations due to factors such as geographic location, population characteristics, and test administration [12,13]. The claimed specificity of TST ranges from 50.65% to 93.60%. The variation in the results emphasizes the impact of variables that can lead to false-positive results, such as the existence of non-tuberculous mycobacteria and previous BCG vaccination [14,15]. Despite its historical significance, the TST’s utility can be limited by false positives in vaccinated individuals and false negatives in immunocompromised patients [16]. The feasibility and cost-effectiveness of TST make it a viable option in high tuberculosis burden countries [17].

QuantiFeron-TB GIT and QuantiFeron-TB Gold Plus

IGRAs, including the QFT-GIT and QFT-Plus, have gained prominence for their enhanced specificity and reduced dependence on patient factors compared to the TST [18,19]. The QFT-TB GIT test shows a sensitivity ranging from 20.90% to 97.74% and a specificity between 42.80% and 97.45% [1,20]. The QFT-Plus has demonstrated a sensitivity of up to 91.10% and a specificity of up to 98.86% [21]. The high specificity of these tests makes them particularly useful in populations where BCG vaccination is prevalent, reducing the likelihood of false positives seen with the TST [22]. However, the sensitivity of these assays can be influenced by the disease stage and the presence of other health conditions [23]. For instance, lower sensitivity in certain populations may necessitate supplementary testing or different diagnostic approaches [24].

T-Spot TB

The T-Spot TB test, another type of IGRA, is noted for its high sensitivity (92%-97%) and specificity (94%-98%) [25,26]. The high specificity makes it a reliable test for distinguishing between latent tuberculosis and other conditions that might cause elevated TST results [27]. The high sensitivity and specificity of the T-Spot TB test contribute to its strong performance in various settings, including those with high BCG vaccination rates or complex clinical presentations [28].

CLIA-IGRA

The CLIA-IGRA test stands out with a perfect sensitivity of 100% and a high specificity of 95.57% [29]. This exceptional performance suggests its potential as the most accurate diagnostic tool among those reviewed [30]. Its high NPV (99.80%) is particularly noteworthy, indicating its effectiveness in ruling out latent tuberculosis in individuals with a negative result [31]. However, the practicality and cost of the CLIA-IGRA may limit its widespread use compared to other, more accessible tests [32,33]. Lack of data also limits the complete practical application of this test in a high tuberculosis burden setup.

HBHA-Induced IFN-γ Release Assay

The HBHA-induced IGRA shows promising performance with a sensitivity of 86.4% and specificity of 82.50% [34]. This test is a good choice in clinical settings because it strikes a balance between sensitivity and specificity, even though its sensitivity may not be as good as that of the CLIA-IGRA [32-36].

## Conclusions

Every diagnostic test for latent tuberculosis has specific benefits and drawbacks. The best sensitivity and specificity are provided by the CLIA-IGRA test, which makes it a great option for precise latent tuberculosis diagnosis. Nonetheless, one must take into account pragmatic factors such as cost, accessibility, and gaps in supporting research. There is still use in the TST and QuantiFeron assays, especially in environments with high tuberculosis burden. Several considerations, including test availability, cost, patient demographics, and the existence of coexisting illnesses, should influence the diagnostic test selection. To enhance latent tuberculosis diagnosis and treatment, future studies should pursue the improvement of current tests and investigate novel diagnostic technologies.

To accurately diagnose and effectively manage latent tuberculosis, a customized strategy combining high-performing testing with clinical judgment is necessary. The capacity to identify and treat latent tuberculosis more successfully will likely continue to improve with future developments and breakthroughs in diagnostic technologies.

## Appendices

**Table 3:** Preferred Reporting Items for Systematic Reviews and Meta-Analyses checklist.

	1	Identify the report as a systematic review	Abstract
Abstract	2	See the PRISMA 2020 for Abstracts checklist	Introduction
Rationale	3	Describe the rationale for the review in the context of existing knowledge	Methods
Objectives	4	Provide an explicit statement of the objective(s) or question(s) the review addresses	Methods
Eligibility criteria	5	Specify the inclusion and exclusion criteria for the review and how studies were grouped for the syntheses	Methods
Information sources	6	Specify all databases, registers, websites, organisations, reference lists and other sources searched or consulted to identify studies. Specify the date when each source was last searched or consulted	Methods
Search strategy	7	Present the full search strategies for all databases, registers and websites, including any filters and limits used	Methods
Selection process	8	Specify the methods used to decide whether a study met the inclusion criteria of the review, including how many reviewers screened each record and each report retrieved, whether they worked independently, and if applicable, details of automation tools used in the process.	Methods
Data collection process	9	Specify the methods used to collect data from reports, including how many reviewers collected data from each report, whether they worked independently, any processes for obtaining or confirming data from study investigators, and if applicable, details of automation tools used in the process	Methods
Data items	10a	List and define all outcomes for which data were sought. Specify whether all results that were compatible with each outcome domain in each study were sought (e.g. for all measures, time points, analyses), and if not, the methods used to decide which results to collect	Methods
	10b	List and define all other variables for which data were sought (e.g. participant and intervention characteristics, funding sources). Describe any assumptions made about any missing or unclear information	Methods
Study risk of bias assessment	11	Specify the methods used to assess risk of bias in the included studies, including details of the tool(s) used, how many reviewers assessed each study and whether they worked independently, and if applicable, details of automation tools used in the process	Methods
Effect measures	12	Specify for each outcome the effect measure(s) (e.g. risk ratio, mean difference) used in the synthesis or presentation of results	Methods
Synthesis methods	13a	Describe the processes used to decide which studies were eligible for each synthesis (e.g. tabulating the study intervention characteristics and comparing against the planned groups for each synthesis	Methods
	13b	Describe any methods required to prepare the data for presentation or synthesis, such as handling of missing summary statistics, or data conversions	Methods
	13c	Describe any methods used to tabulate or visually display results of individual studies and syntheses	Methods
	13d	Describe any methods used to synthesize results and provide a rationale for the choice(s). If meta-analysis was performed, describe the model(s), method(s) to identify the presence and extent of statistical heterogeneity, and software package(s) used	No
	13e	Describe any methods used to explore possible causes of heterogeneity among study results (e.g. subgroup analysis, meta-regression)	Methods
	13f	Describe any sensitivity analyses conducted to assess robustness of the synthesized results	No
Reporting bias assessment	14	Describe any methods used to assess risk of bias due to missing results in a synthesis (arising from reporting biases)	Results
Certainty assessment	15	Describe any methods used to assess certainty (or confidence) in the body of evidence for an outcome	Methodology
Study selection	16a	Describe the results of the search and selection process, from the number of records identified in the search to the number of studies included in the review, ideally using a flow diagram	Results
	16b	Cite studies that might appear to meet the inclusion criteria, but which were excluded, and explain why they were excluded	Results
Study characteristics	17	Cite each included study and present its characteristics	Results
Risk of bias in studies	18	Present assessments of risk of bias for each included study	Results
Results of individual studies	19	For all outcomes, present, for each study: (a) summary statistics for each group (where appropriate) and (b) an effect estimate and its precision (e.g. confidence/credible interval), ideally using structured tables or plots	Results
Results of syntheses	20a	For each synthesis, briefly summarise the characteristics and risk of bias among contributing studies	Results
	20b	Present results of all statistical syntheses conducted. If meta-analysis was done, present for each the summary estimate and its precision (e.g. confidence/credible interval) and measures of statistical heterogeneity. If comparing groups, describe the direction of the effect	Results
	20c	Present results of all investigations of possible causes of heterogeneity among study results	Discussion
	20d	Present results of all sensitivity analyses conducted to assess the robustness of the synthesized results	Discussion
Reporting biases	21	Present assessments of risk of bias due to missing results (arising from reporting biases) for each synthesis assessed	No
Certainty of evidence	22	Present assessments of certainty (or confidence) in the body of evidence for each outcome assessed	Discussion
Discussion	23a	Provide a general interpretation of the results in the context of other evidence	Discussion
	23b	Discuss any limitations of the evidence included in the review	
	23c	Discuss any limitations of the review processes used	
	23d	Discuss implications of the results for practice, policy, and future research	Not registered
Registration and protocol	24a	Provide registration information for the review, including register name and registration number, or state that the review was not registered	Nil
	24b	Indicate where the review protocol can be accessed, or state that a protocol was not prepared	No funding
	24c	Describe and explain any amendments to information provided at registration or in the protocol	No
Support	25	Describe sources of financial or non-financial support for the review, and the role of the funders or sponsors in the review	Data used for analysis from Figures 1-13
Competing interests	26	Declare any competing interests of review authors	No
Availability of data, code and other materials	27	Report which of the following are publicly available and where they can be found: template data collection forms; data extracted from included studies; data used for all analyses; analytic code; any other materials used in the review	Data extracted - Articles added in References
